# Studies on the Interaction Mechanism of Pyrene Derivatives with Human Tumor-Related DNA

**DOI:** 10.3390/molecules171214159

**Published:** 2012-11-28

**Authors:** Li Li, Jia Lu, Chongzheng Xu, Huihui Li, Xiaodi Yang

**Affiliations:** Jiangsu Key Laboratory of New Power Batteries, Jiangsu Key Laboratory of Biofunctional Materials, School of Chemistry and Materials Science, Nanjing Normal University, Nanjing 210097, China

**Keywords:** pyrene derivatives, oncogene, tumor suppressor gene, DNA interaction, PAGE

## Abstract

Pyrene derivatives can be carcinogenic, teratogenic and mutagenic, thus having the potential to cause malignant diseases. In this work, the interactions of two selected pyrene derivatives (1-OHP and 1-PBO) and human tumor-related DNA (*p53* DNA and *C-myc* DNA) are investigated by spectroscopic and non-native polyacrylamide gel electrophoresis (PAGE) methods. Using fluorescence spectrometry and circular dichroism (CD), DNA interactions of pyrene derivatives are confirmed to occur mainly via the groove binding mode supported by the intercalation into the base pairs of DNA. There is an obvious binding order of pyrene derivatives to the targeted DNA, 1-OHP > 1-PBO. The binding constants of 1-OHP are 1.16 × 10^6^ L·mol^−1^ and 4.04 × 10^5^ L·mol^−1^ for *p53* DNA and *C-myc* DNA, respectively, while that of 1-PBO are only 2.04 × 10^3^ L·mol^−1^ and 1.39 × 10^3^ L·mol^−1^ for *p53* DNA and *C-myc* DNA, respectively. Besides, the binding of pyrene derivatives to *p53* DNA is stronger than that for *C-myc* DNA. CD and PAGE results indicate that the binding of pyrene derivatives can affect the helical structures of DNA and further induce the formation of double-chain antiparallel G-quadruplex DNA of hybrid G-rich sequences.

## 1. Introduction

Pyrene and its derivatives contain aromatic conjugated systems which consist of four fused benzene rings. As polycyclic aromatic hydrocarbons derivatives (PAHs), they are well known as carcinogenic, teratogenic and mutagenic, with bio-accumulative effects [1,2]. Pyrene and its derivatives have been used commercially as dyes and dye precursors. Among them, it is well known that hydroxypyrene is a major metabolite of pyrene in mammals [3]. Hydroxypyrene derivatives have good performance in making synthetic resins, as well as disperse dyes and optical pressure-sensitive paints. Results of animal experiments have confirmed that they are toxic to the kidneys and the liver, though not as serious as benzopyrene [4,5,6]. 

Cancer is a leading cause of death worldwide, so there is no doubt that the treatment and prevention of cancer are among the most critical issues in global health. More and more evidence has indicated that the formation of tumors is due to multiple factors with oncogene activation and anti-oncogene inactivation. *C-myc *oncogene is responsible for promoting cell growth and proliferation, acting as one of the key genes for the malignant transformation of cells [7]. Over expression of *C-myc *gene is crucial for certain types of genomic instability, such as gene amplification in human cancer cells. Tumor suppressor genes are responsible for the inhibition of cell growth or the regulation of cell division. For instance, inactivation of the *p53* tumor suppressor is a frequent event in tumorigenesis. Losing of function or abnormal expression of *p53* tumor suppressor gene is found in nearly half of all cancer cells [8,9].

**Figure 1:**
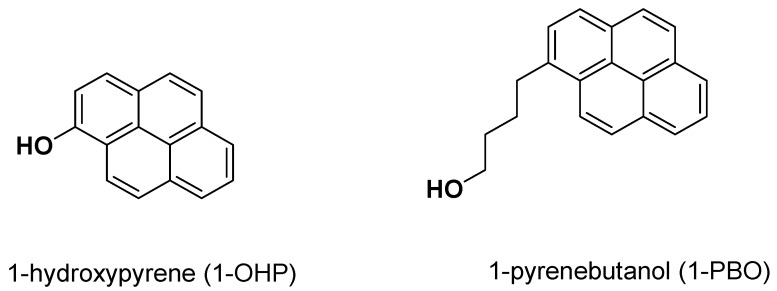
Structures of pyrene derivatives.

It has been considered that the necessary step in the activation process of genotoxic carcinogens, perhaps after the metabolic activation, is their DNA interaction [10]. Due to gene damage or mutation, tumor cells would lose control and continue to grow [11,12]. Thus, we choose two double-strand DNA sequences in the promoter regions of *C-myc* oncogene and *p53* tumor suppressor gene as targets. In this work, we selected two pyrene derivatives, 1-hydroxypyrene (1-OHP) and 1-pyrenebutanol (1-PBO) (Figure 1), to investigate the interactions with *p53* DNA and *C-myc* DNA by steady and transient state fluorescence spectrometry, circular dichroism (CD) and non-native polyacrylamide gel electrophoresis (PAGE) [13,14,15]. The purpose was to explore the relationships between the molecular structures of pyrene derivatives and their DNA interaction mechanisms for biological and environmental assessments.

## 2. Results and Discussion

### 2.1. Steady State Fluorescence Studies

**Figure 2:**
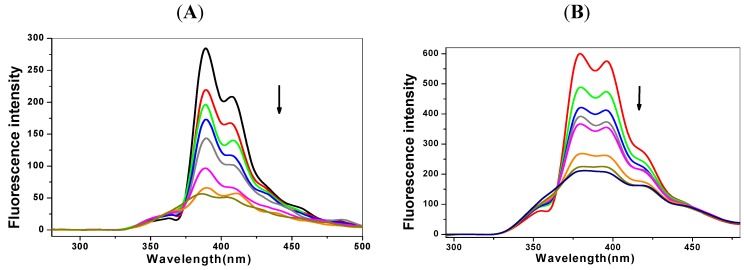
Fluorescence spectra of the pyrenederivatives solution (1 μmol/L) with the addition of *p53* DNA (0-20 μmol/L). (**A**) 1-OHP and (**B**) 1-PBO. The arrows show the fluorescence changes at peak wavelength with the DNA concentration increased.

Fluorescence analysis, with its excellent sensitivity and accuracy, is widely used to study the interactions between DNA and drugs or poisonous molecules. Pyrene derivatives have fluorescence, shown in Figure 2, because of their large conjugated system and rigid planar structures. Pyrene groups in the excited state themselves could form charge transfer complexes, known as excited dimers. Therefore, pyrene derivatives present two fluorescence peaks, including the red shift emission of the dimer compared to the monomer. 

The fluorescence spectra here are used to estimate the binding mode and the binding abilities of pyrene derivatives to DNA. After mixing with DNA at increasing concentrations, the pyrene derivatives solution shows a gradually weakening in fluorescence, as shown in Figure 2 for *p53* DNA. When the molar ratio of DNA and pyrene derivatives is 20:1, the quenching is about 80%. This result shows that there may be energy and electron transfers occurring between pyrene derivatives and DNA, leading to the fluorescence quenching. 

Although changes in the fluorescence of the pyrene derivatives solution in the presence of DNA act as evidence of interactions, it cannot be a direct proof of the binding mode of pyrene derivatives and DNA. It may because hydrophobic interactions between pyrene derivatives and DNA change the microenvironment of pyrene derivatives, and this hinders the electron transition [16]. The Stern–Volmer equation [Equation (1)] [17] and the Scatchard equation [Equation (2)] [18] can be used to describe the fluorescence quenching process of pyrene derivatives as follows:

F_0_/F* = *1*+ K*_q_*τ*_0_ [DNA]* = *1 *+ K*_SV_ [DNA] (1)

log[(F_0_ − F)/F] *=* log*K_b_ + n* log[DNA] (2)

where F_0_ is the fluorescence intensity of pyrene derivatives. F is the fluorescence intensity of pyrene derivatives with the addition of the quenching agent, DNA. [DNA] is the concentration of DNA added. τ_0_ is the average life expectancy of the fluorescent quencher molecule. For a biomacromolecule, τ_0_ is 10 ns on average [19]. *K_SV_* is the linear Stem-Volmer constant, which can be calculated from Equation (1). *K_q_* is the rate constant of the quenching process, which is equal to *K_SV_* divided by τ_0_. *K_b_* is the binding constant and *n* is the binding number of DNA to pyrene derivatives, which can be calculated from Equation (2).

The calculated results, *K*_q_, *K_SV_*, *K*_b_ and *n*, are available in Table 1. First, the quenching rates of DNA, *K*_q_, are all above 10^12^ L·mol^−1^℘s^−1^, indicating that their interactions with pyrene derivatives involve static quenching [20]. Second, the results suggest that the values of *K_b_* of pyrene derivatives to *p53* DNA are commonly larger than that for *C-myc* DNA, suggesting the binding specificity of pyrene derivatives to *p53* DNA superior to that for * C-myc* DNA. Third, the shorter the side chain of pyrene derivatives, the larger the *K_b_* values are obtained (4.04 × 10^5^ L·mol^−1^ of 1-OHP and 1.39 × 10^3^ L·mol^−1^ of 1-PBO for *C-myc* DNA, respectively). Therefore, for the same target (*C-myc* DNA or *p53* DNA), the order of the binding ability is 1-OHP > 1-PBO.

**Table 1:** Summary of pyrene derivatives—DNA interactions observed by fluorescence spectroscopy.

	*K*_sv_ (×10^5^ L·mol^−1^)	*K*_q_ (×10^13^L·mol^−1^℘s^−1^)	Δ (%) *	*n*	*K*_b_ (L·mol^−1^)
*C-myc* + 1-OHP	1.97	1.97	72.34	1.07	4.04 × 10^5^
*p53* + 1-OHP	2.53	2.53	82.93	1.13	1.16 × 10^6^
*C-myc* + 1-PBO	1.32	1.32	77.34	0.56	1.39 × 10^3^
*p53* + 1-PBO	1.57	1.57	78.38	0.58	2.04 × 10^3^

* represents quenching extent Δ (%) = (F − F_0_)/F_0_ × 100%

### 2.2. The Binding Type of the Binary Complex and Thermodynamic Studies

**Figure 3:**
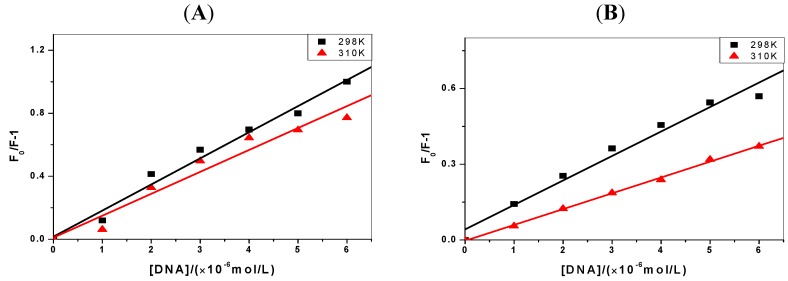
The Stern-Volmer plots of the fluorescence quenching of pyrene derivatives by *C-myc *DNA at 298 K and 310 K (the concentrations of pyrene derivatives are 1 μmol/L, pH = 7.4). (**A**) 1-OHP and (**B**) 1-PBO.

The thermodynamic experiments were performed at room temperature and physiological temperature (298 K and 310 K, respectively). From the results of *C-myc* DNA in Figure 3, we can see that the slope of Stern-Volmer curves decrease as the temperature rises from 298 K to 310 K. That is, the Stern-Volmer constant, *K_SV_*, is inversely proportional to the temperature, which confirms that static quenching of DNA with the pyrene derivatives happens [21]. 

The thermodynamic constants (Gibbs free energy *ΔG*, enthalpy *ΔH*, entropy *ΔS*) could be calculated according to Equations (3–5). The subscripts “1” or “2” in our work refers the condition of 298 K or 310 K, respectively:

ln*K_b1_*/*K_b2_ = *(1/T_1_ − 1/T_2_) ×*ΔH*/R (3)

*ΔG = −*RTln*K_b_* (4)

*ΔG = ΔH − *T*ΔS* (5)

The relevant thermodynamic constants are summarized in Table 2. The negative values of *ΔH* and *ΔS* indicate that pyrene derivatives bind to the DNA mainly via van der Waals forces and hydrogen bonds [22], which are widely considered to make an important contribution to the binding in the minor groove of DNA [16]. Thus, the minor groove binding of pyrene derivatives can be considered. Since the larger magnitude of *ΔG* indicates a greater interaction possibility, for the same target (*C-myc* DNA or *p53* DNA), the order of the binding ability is 1-OHP>1-PBO, and the binding specificity of pyrene derivatives to *p53* DNA is superior to that for * C-myc* DNA. 

**Table 2:** Thermodynamic constants of the interaction of pyrene derivatives with DNA.

	*ΔH*(kJ·mol^−1^)	*ΔG*(kJ·mol^−1^)	*ΔS*(J·mol^−1^·K^−1^)
*C-myc* + 1-OHP	−48.62	−31.98	−55.84
*p53* + 1-OHP	−54.77	−34.60	−67.68
*C-myc *+ 1-PBO	−37.46	−17.93	−65.54
*p53* + 1-PBO	−40.94	−18.88	−70.34

### 2.3. Effect of the Ionic Strength on the Fluorescence Properties

Sodium chloride, NaCl, is used as the ionic strength modifier to determine whether there are electrostatic interactions between DNA and pyrene derivatives. The increased ionic strength of solution can inhibit the electrostatic interactions between DNA and binding molecules [23]. If the enhanced fluorescence of the DNA-pyrene derivatives solution is observed with added NaCl, indicating that the amplitude of the fluorescence quenching of pyrene derivatives binding with DNA is weakened, an electrostatic interaction can be concluded. There are little changes in the extent of quenching of DNA-pyrene derivative solutions in the absence and presence of NaCl (<0.1%), indicating that the pyrene derivatives don’t have electrostatic interaction with DNA. 

### 2.4. Iodide Quenching Studies

**Figure 4:**
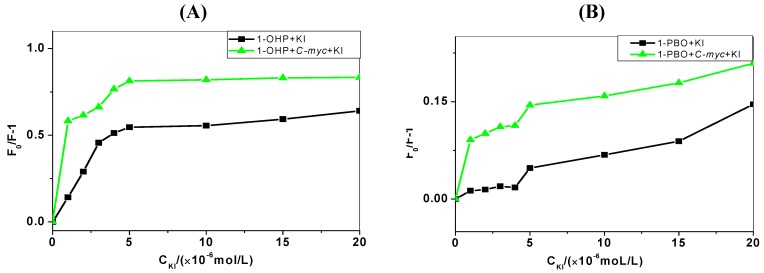
Quenching extents of pyrene derivatives solutions (1 μmol/L) with the addition of KI (0–20 μmol/L) in the absence and presence of *C-myc *DNA (10 μmol/L). (**A**) 1-OHP and (**B**) 1-PBO.

Anionic quenchers, such as potassium iodide (KI) can quench the fluorescence of organic molecules. When the organic molecule binds into the groove region of DNA, I^−^ ions exposed in solution can increase the extent of quenching [24]. For intercalation or electrostatic effect of organic molecules, I^−^ ions will be protected by the phosphate backbone and base pairs of DNA, and the extent of quenching can be reduced. As shown in Figure 4, the quenching extents of pyrene derivatives with the addition of KI in the absence and presence of DNA are denoted by (*F*_0_/*F* − 1). With the increasing concentration of KI, the (*F*_0_/*F* − 1) value gradually increases. When the KI concentration continues to increase over 5 μmol/L, the increase of the (*F*_0_/*F* − 1) value slows down since the reactions reach saturation.

This shows significantly that in the presence of KI, the quenching extent of pyrene derivatives binding to *C-myc* DNA is increased, indicating the existence of the groove binding mode of pyrene derivatives to DNA. 

### 2.5. Competitive Binding between EB and Pyrene Derivatives for DNA

Ethidium bromide (EB) has weak fluorescence in solution. When EB intercalates base pairs of the DNA double helix, its fluorescence intensity can increase 50–100 fold. If the conformation of the duplex DNA changes, or there are organic molecules competing with EB for the DNA interaction, EB can be freed from DNA complexes, causing fluorescence quenching. Therefore, the fluorescence changes of the EB-DNA system in the absence and presence of organic molecules can be used to identify the DNA intercalation [25,26,27,28,29]. Here, EB is used as a fluorescent probe on interactions between organic molecules and DNA. As shown in Table 3, the fluorescence change of the EB-DNA system is represented by *R* (%) = (F_EB-P_ − F_EB_)/F_EB_ × 100%, where F_EB_ and F_EB-P_ represent for the EB-DNA’s fluorescence intensity in the absence and presence of pyrene derivatives, respectively.

**Table 3:** Changes in quenching extents of EB-DNA system in the absence and presenceof pyrene derivatives.

Solution	*R* (%)
*C-myc* + 1-OHP	−5.46
*p53* + 1-OHP	−13.17
*C-myc *+ 1-PBO	−8.74
*p53* + 1-PBO	−8.85

*R* (%) values from −5.46% to −13.17% indicate that both 1-OHP and 1-PBO can compete with EB as weak intercalating agents in DNA interactions. Comparing the *R* (%) values of 1-OHP, 1-OHP is intercalates easier into the base pairs of *p53* DNA than those of *C-myc* DNA. As for 1-PBO, approximate values of *R* (%) (−8.74% and −8.85% for *C-myc* and *p53 *DNA, respectively) show that there is no selectivity of DNA intercalation.

### 2.6. CD Spectra Characteristics

**Figure 5:**
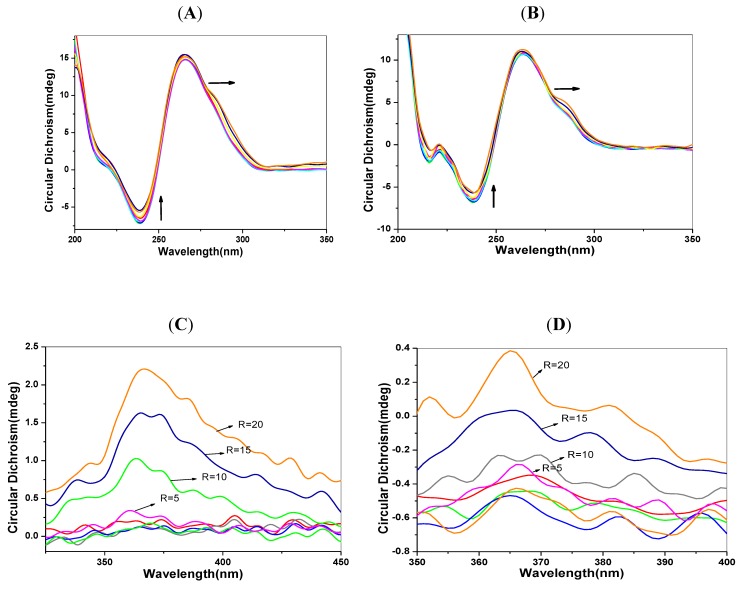
CD and ICD spectra of DNA solutions (5 μmol/L) with the addition of 1-PBO (0–20 μmol/L). (**A**) CDspectra of *p53* DNA; (**B**) CDspectra of *C-myc* DNA; (**C**) ICDspectra of *p53* DNA and (**D**) ICDspectra of *C-myc* DNA.

Circular dichroism spectra can provide conformational information about biomacromolecules. The positive peak at 260–280 nm is the signal of accumulation of DNA base pairs, and the negative peak at 245 nm corresponds to the double helix structure of B-type DNA [30]. The parallel G-quadruplex has a positive peak at 265 nm and a negative peak at 245 nm. The anti-parallel G-quadruplex has apositive peak at 295nm and a negative peak at 265 nm. A shoulder peak at 280 nm indicates polyploidy formation [31,32]. Organic molecules without CD signals may have the induced signals (ICDs) observed when they bind to DNA. It is generally believed that intercalating agents often show a slight negative ICD signal, while those in the groove binding mode often appear as a positive ICD signal [33,34]. Figure 5 shows the CD and ICD spectra of DNA solutions with the increased concentration of 1-PBO. An obvious positive ICD signal appears in Figure 5C, indicating that 1-PBO binds into the groove of the duplex DNA. In Figure 5D, the positive ICD signal of *p53* DNA, weaker than that of *C-myc* DNA, suggesting that 1-PBO plays a stronger role on the groove binding of *p53* DNA than that for *C-myc* DNA. As shown in Figure 5A,B, when mixed with 1-PBO, the negative peak of DNA at 245 nm in CD spectra is reduced, suggesting that the O atom of 1-PBO may form hydrogen bonds with base pairs in the groove of DNA. 

Besides, with increased concentration of 1-PBO, a shoulder peak appears at about 290 nm indicating that there may be the formation of the anti-parallel G-quadruplex of the G-rich DNA sequence. As for the 1-OHP, apart from the similar reduced negative peak at 245 nm, there is an apparent reduced positive peak at 265 nm in the CD spectra of DNA, due to the intercalation of 1-OHP. This result confirms that the binding of pyrene derivatives can affect the normal conformation of the duplex DNA. Combined with the results of iodide quenching and thermodynamic studies, it can be concluded that the DNA interactions of pyrene derivatives are dominated by groove binding. Besides, results of EB competition experiments confirm the existence of intercalation in the binding of pyrene derivatives into DNA base pairs.

### 2.7. Transient Fluorescence Studies

**Figure 6:**
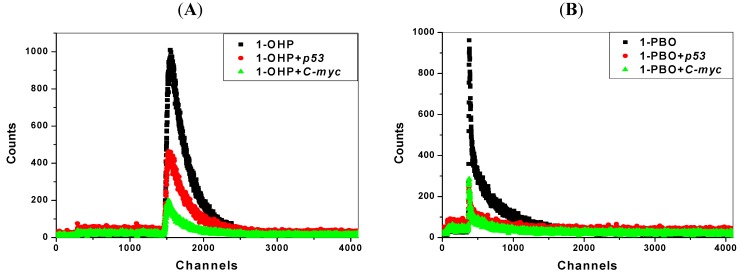
Transient fluorescence spectra of pyrene derivatives (1 μmol/L) in the absence and presence of DNA (10 μmol/L).(**A**) 1-OHP and (**B**) 1-PBO.

Transient fluorescence spectroscopy has recently become a critical tool in the biochemical and biophysical field [35]. The transient fluorescence experiments on lifetimes are used to discuss the binding modes of pyrene derivatives with *p53* DNA and *C-myc* DNA, as shown in Figure 6 and Table 4. The lifetime I of 1-OHP is observed as 13.8 ns. With the addition of DNA, its lifetime changes little. Consistent with results in steady state fluorescence quenching experiments, this indicates that static quenching dominates in the DNA-1-OHP binding process [36]. Due to the conformational complexity of 1-PBO with the long side-chain, there are two lifetimes, 90.7 ns and 4.90 ns. The distribution of the longer one is related to the DNA-bound structure of 1-PBO and the shorter one corresponds to the unbound structure. Besides, the magnitude of lifetime often reflects the variations in fluorophore-DNA interactions resulting from changes in the structure of the organic molecules or DNA molecules and environmental changes [37,38]. Since the unbound molecules have characteristic fluorescence lifetimes depending on their circumstances, the changes shown with the unbound lifetime component suggest they are sensitive to the changes in molecular environment [39]. According to the results, the lifetime of DNA-bound component changes little, which confirms the static quenching. The average lifetime of 1-PBO with DNA interaction is shorter than that of unbound 1-PBO, suggesting that 1-PBO does affect the DNA double helix structure, similar with some groove binding and intercalation agents in previous studies [38,40]. Moreover, combining the Stern-Volmer fluorescence data with lifetime measurement data discussion, we again confirmed the static quenching that existed in the interaction of pyrene derivatives with the two kinds of DNA.

**Table 4:** The calculated lifetime of pyrene derivatives in the absence and presence of DNA.

Solution	Lifetime(ns) *	Solution	Lifetime(ns) **		
			T1	T2	Average
1-OHP	13.8	1-PBO	90.7	4.90	40.6
1-OHP + *p53*	14.0	1-PBO + *p53*	86.9	3.79	35.1
1-OHP + *C-myc*	13.6	1-PBO + *C-myc*	87.7	3.96	28.2

* represents the lifetime calculated using a mono-exponential decay function; ** represents the lifetime calculated using a bi-exponential decay function.

### 2.8. PAGE Studies

**Figure 7:**
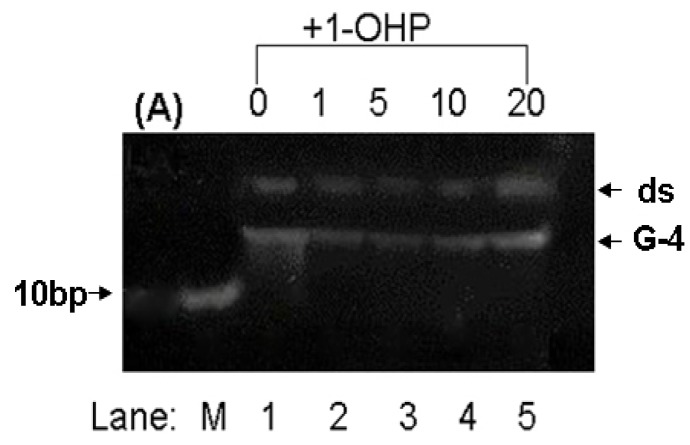
PAGE image of *p53* DNA with the addition of 1-OHP solution in different molar ratio. (Lane M): DNA marker, (Lane 1–5): the molar ratio of DNA and 1-OHP is 1:0, 1:1, 1:5, 1:10 and 1:20, respectively.

Non-native polyacrylamide gel electrophoresis (PAGE) can be used for the separation and purification of biological macromolecules [41,42]. Especially, high concentration PAGE has a higher resolution in the separation of short DNA fragments. The effect of 1-OHP on the structure of *p53* DNA is shown in Figure 7. Since G-quadruplex DNA of two hybrid G-rich sequences (referred as G-4) has more charge and a more tight structure than double-strand DNA (referred to as ds), G-4 will have a faster mobility rate than ds. As shown in Figure 7, the double-strand strips of *p53 *DNA slightly diffuse in the presence of 1-OHP, indicating that 1-OHP doesn’t affect the stability of the helix structure of double-strand DNA. However, as the molar ratio of 1-OHP and DNA increases, the G-4 strip becomes brighter, indicating the formation trend of G-quadruplex DNA, which is also confirmed by the CD results.

## 3. Experimental

### 3.1. Apparatus

Fluorescence measurements were performed with a LS50B spectrofluorimeter (Perkin Elmer, Massachusetts, MA, USA). Transient fluorescence spectra were measured on a FM-4P-TCSPC Spectrofluorometer (Horiba Jobin Yvon, Paris, France). Circular dichroism spectra were recorded with a Chirascan circular dichroism spectrophotometer (Applied Photophysics, Leatherhead, UK). Non-native polyacrylamide gel electrophoresis was performed in a PROTEAN II xi Cell (Bio-Rad, Hercules, CA, USA), and the PAGE image was captured by GelDoc-It TS Imaging System (UVP, Citrus Heights, CA, USA). PH measurements were carried out with a Seven Multi pH digital pH-meter (Mettler Toledo, Shanghai, China) with a combined glass-calomel electrode. An electronic thermostat water-bath (Changzhou Guohua Medical Instrument Company, Changzhou, China) was used for controlling the temperature.

### 3.2. Reagents

*p53* gene P1 promoter fragment (ss1:5′-CCTCCTCCCCAACTCC-3′, ss2:3′-GGAGGAGGGGTT-GAGG-5′); *C-myc* gene promoter of NHE III1 fragment (ss1:5′-GGGAGGGTGGGGAAGG-3′, ss2:3′-CCCTCCCACCCCTTCC-5′). Single-stranded oligonucleotides were diluted with 100 mmol/L Tris-HCl buffer solution (pH = 7.4). For duplex DNA, two complementary single-stranded oligonucleotides were mixed in equimolar proportions, annealed at 85 °C and slowly cooled to room temperature to form of the duplex (ds) at 500 μmol/L: A stock solution (10 mmol·L^−1^) of pyrene derivatives (AccuStandard, USA) was prepared by dissolving its crystals in methanol (Sigma-Aldrich, St Louis, MO, USA), and then diluted to different concentrations. Ethidium bromide (EB, AccuStandard, Palo Alto, CA, USA) solution, NaCl solution and KI solution (500 μmol·L^−1^ and 100 μmol·L^−1^) were all prepared by dissolving its crystals in water. SYBR Green I (Unique, Beijing, China); DNA Ladder, Ultra Low Range (Fermentas, Burlington, ON, Canada); 6 × Orange DNA Loading Dye (Fermentas). Pure 18-MΩ MilliQ water was used for preparation of all solutions.

### 3.3. General Procedures

#### 3.3.1. Fluorescence Quenching Experiments

Pyrene derivatives solution (500 μL, 1 μmol/L) was mixed with the duplex DNA (0–40.0 μL), and allowed to stand for 5 min to equilibrate. The excitation wavelength was 275 nm for all cases with an excitation and emission band pass (slit) of 5 nm. This experiment was undertaken at different temperatures (298 K and 310 K).

#### 3.3.2. Ionic Strength Experiments

Pyrene derivatives (500 μL, 1 μmol/L) and DNA (10.0 μL, 500 μmol/L) were premixed, and then NaCl (0–40.0 μL) was added and the mixtures left standing for 5 min to equilibrate. The excitation wavelength was 275 nm for all cases with an excitation and emission band pass (slit) of 5 nm. 

#### 3.3.3. KI Quenching Experiments

Pyrene derivatives solution (500 μL, 1 μmol/L) was mixed with KI (0–40.0 μL). In the comparison experiment, pyrene derivatives (500 μL, 1 μmol/L) and DNA (10 μL 500 μmol/L) were premixed, then mixed with KI (0–40.0 μL) and left standing for 5 min to equilibrate. In both cases, the excitation wavelength was 275 nm with an excitation and emission band pass (slit) of 5 nm. 

#### 3.3.4. EB Competition Experiments

DNA (500 μL 1 μmol/L) and EB (10.0 μL, 500 μmol/L) were premixed, and then pyrene derivatives (0–40.0 μL) were added and the mixtures left standing for 5 min to equilibrate. The excitation wavelength was 480 nm with an excitation and emission band pass (slit) of 15 nm. 

#### 3.3.5. Circular Dichroism Spectra Experiments

Pyrene derivatives (0–40.0 μL) were added to DNA (500 μL 10 μmol/L) in molar ratios ranging from 1:0 to 1:20, and left standing for 5 min to equilibrate. All spectra were recorded in the range of 200–500 nm. A spectrum of buffer solution was recorded and subtracted from the spectra of DNA and pyrene derivatives-DNA complexes.

#### 3.3.6. Transient Fluorescence Experiments

Pyrene derivatives (500 μL 1 μmol/L) and DNA (10.0 μL, 500 μmol/L) were mixed together in a 1:10 molar ratio. Then the time-resolved fluorescence spectra of pure pyrene derivatives and pyrene derivatives-DNA solutions were measured. The experimental conditions were the same as those of the fluorescence quenching experiments.

#### 3.3.7. PAGE Experiments

To prepare pure DNA and pyrene derivatives the DNA samples were premixed with DNA Loading Dye to a concentration of about 1 mg/mL. After the 16 × 16 × 0.1 cm gel (20%, 29:1 mono:bis ratio) formed, the samples (10.0 μL) were added to each lane. Electrophoresis was carried out under a constant voltage of 100 V for 12 h under 1 × TBE buffer, stained by SYBR Green I for 30 min, and then the gel image was captured.

## 4. Conclusions

In this work, fluorescence spectroscopy, CD and non-native PAGE methods are used to evaluate interactions of two pyrene derivatives (1-OHP and 1-PBO) with human tumor-related DNA (*p53* and *C-myc* DNA), respectively. Fluorescence and CD spectra indicate that hydroxypyrene binds to DNA in the groove and intercalated stacked DNA base pairs to strengthen their binding. Through thermodynamic constants, it is concluded that van der Waals forces and hydrogen bonds exist between the pyrene derivatives and DNA. The spectroscopic results show their obvious binding behavior to the targeted DNA with the order of binding constants: 1-OHP > 1-PBO, and their binding stoichiometry. In addition, the binding ability of pyrene derivatives to *p53* DNA is observed to be superior to that for* C-myc* DNA. CD and PAGE results show that DNA interactions of pyrene derivatives can lead to the conformational changes of the duplex DNA, and further induce the antiparallel G-quadruplex formation of G-rich sequences. It is indeed observed that pyrene derivatives have non-covalent interactions with the critical duplex DNA sequences of human oncogenes and tumor suppressor genes, which may be the fundamental reason for abnormal expression of the tumor-related genes in the human body.
